# Treatment patterns and outcomes in patients with metastatic gastric cancer receiving third-line chemotherapy: A population-based outcomes study

**DOI:** 10.1371/journal.pone.0198544

**Published:** 2018-06-07

**Authors:** In Sil Choi, Mihong Choi, Ju Hyun Lee, Jee Hyun Kim, Koung Jin Suh, Ji Yun Lee, Beodeul Kang, Ji-Won Kim, Se-Hyun Kim, Jin Won Kim, Jeong-Ok Lee, Yu Jung Kim, Soo-Mee Bang, Jong Seok Lee, Keun-Wook Lee

**Affiliations:** 1 Department of Internal Medicine, Seoul National University College of Medicine, Seoul Metropolitan Government Seoul National University Boramae Medical Center, Seoul, Republic of Korea; 2 Department of Internal Medicine, Seoul National University College of Medicine, Seoul National University Bundang Hospital, Seongnam, Republic of Korea; University of Texas MD Anderson Cancer Center, UNITED STATES

## Abstract

**Purpose:**

There is limited data on third-line chemotherapy in patients with metastatic gastric cancer (MGC). This study was conducted to assess third-line treatment patterns, outcomes, and clinical parameters related to survival outcomes in patients with MGC.

**Methods:**

Using the Korean Health Insurance Review and Assessment Service (HIRA) database, a nationwide population-based outcomes study was conducted. From the HIRA database, patients newly diagnosed in 2010 with MGC were identified (N = 1,871), and of these, 229 patients who had received third-line chemotherapy were finally selected for this study.

**Results:**

Prior to third-line chemotherapy, more than 90% of patients received fluoropyrimidine and platinum, and 43.7% and 40.6% received taxane and irinotecan, respectively. Various third-line chemotherapy regimens containing taxane (docetaxel or paclitaxel), irinotecan, or oxaliplatin were prescribed. The median overall survival (OS) of all patients receiving third-line chemotherapy was 4.4 months. The median time from the start date of first-line chemotherapy to the start date of third-line chemotherapy (TF1T3) was 9.5 months, and a longer TF1T3 was the only factor that was significantly associated with an increased OS. The median OS of patients who had received fluoropyrimidine, platinum, and taxane followed by third-line irinotecan-based therapy was similar to that of patients who had received fluoropyrimidine, platinum, and irinotecan followed by third-line taxane-based therapy (*p* = 0.894).

**Conclusion:**

In patients with MGC receiving third-line chemotherapy, TF1T3 was the only significant factor associated with OS. The sequence of using taxane and irinotecan as subsequent therapy after first-line failure was not shown to impact survival outcome.

## Introduction

Although the incidence of gastric cancer (GC) has been declining over the past several decades, it remains the third most common cause of cancer death worldwide [1]. In Korea, GC has the second highest incidence of all cancers and it is the third leading cause of cancer death [2]. For patients with metastatic or recurrent GC, palliative chemotherapy prolongs overall survival (OS) and provides significant palliation of symptoms compared to the best supportive care (BSC) alone [3–5]. Although fluoropyrimidine and platinum combination chemotherapy is widely recognized as the standard first-line treatment, the median progression-free survival (PFS) has been reported to be 4–7 months, and nearly all patients will eventually develop progressive disease following first-line chemotherapy [6–10]. Several recent phase III studies have shown that second-line chemotherapy provides an improvement in OS when compared to BSC [11–15]. In these studies, three cytotoxic chemotherapeutic agents (paclitaxel, docetaxel, and irinotecan) and one anti-vascular endothelial growth factor receptor antibody (ramucirumab) were shown to be associated with significant reductions in the risk of death. Therefore, second-line chemotherapy is currently considered the standard of care in patients with metastatic or recurrent GC after first-line failure. Nevertheless, many patients receiving second-line treatment fail to achieve response and even in responders, the duration of response is as short as a few months. While it is common practice to offer further lines of chemotherapy after second-line failure, especially in Eastern Asian countries, there is limited data on the efficacy of third-line chemotherapy in patients with metastatic or recurrent GC. Since some patients with good performance status (PS) are candidates for and may benefit from third-line therapy, it is essential to identify the subset of patients with the greatest likelihood of benefitting from third-line chemotherapy.

To the best of our knowledge, nationwide or population-based studies reflecting real-world treatment patterns and outcomes in patients with metastatic or recurrent GC receiving third-line chemotherapy are very scarce. To define the potential role of third-line chemotherapy in these patients, we conducted this study to assess third-line treatment patterns, outcomes, and clinical parameters related to survival outcomes.

## Materials and methods

### Study population

The Health Insurance Review and Assessment Service (HIRA) in Korea, is a government-affiliated organization created to build an accurate claims review and medical quality assessment system. Hospitals and clinics submit inpatient and outpatient claims data on spent cost covered by National Health Insurance or Medical Aid programs to the HIRA for reimbursement. Submitted data includes diagnostic codes, demographic information, medical practice items that were employed for diagnosis and treatment, and prescribed medications. Since the HIRA database includes all claims data submitted from all hospitals and clinics in Korea, a population-based study is possible.

**Fig 1:**
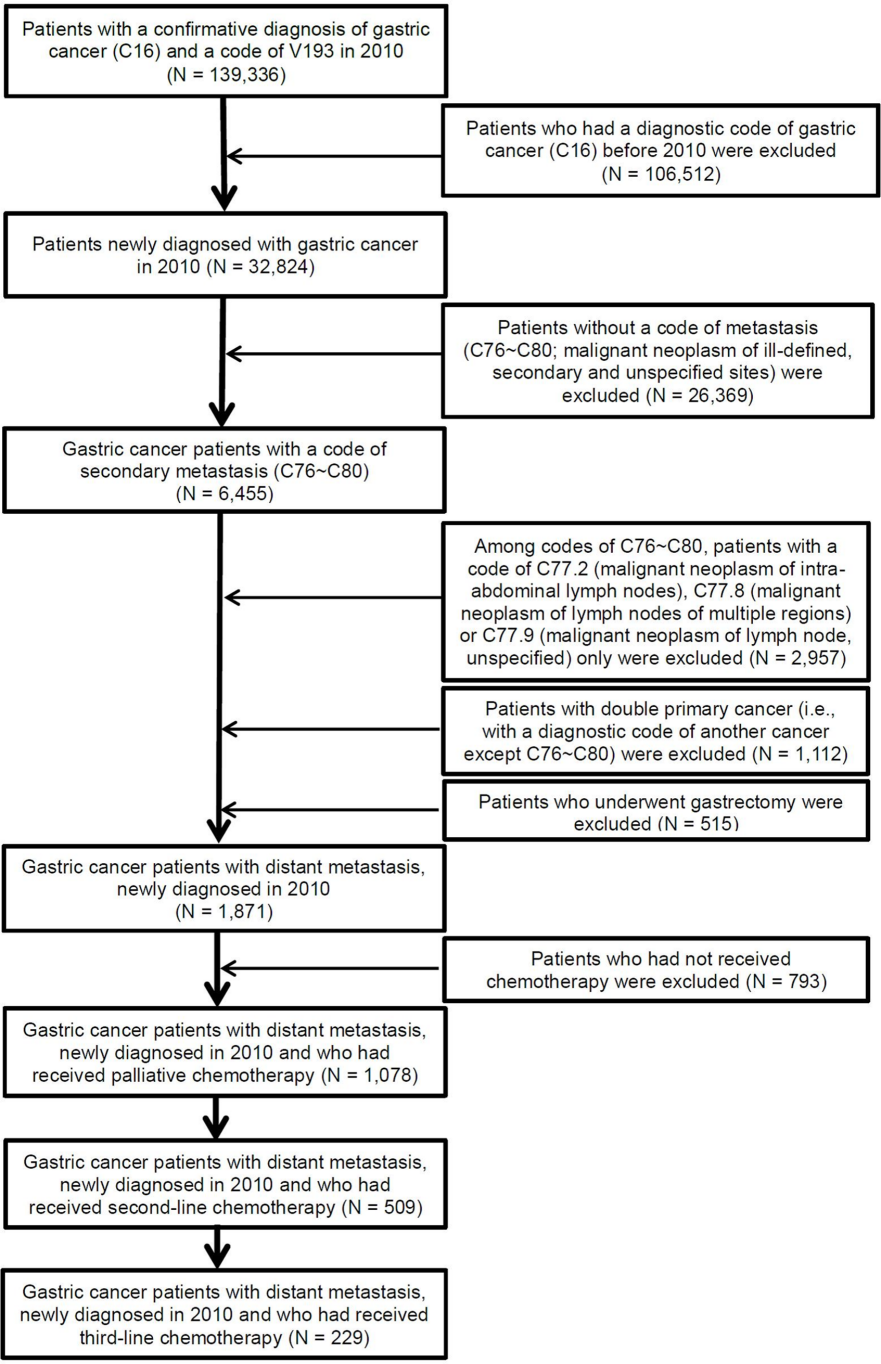
Study population and the process of case identification.

We identified 1,871 patients who were newly diagnosed with metastatic gastric cancer (MGC, stage IV) in 2010 (Fig 1). The process of identification of these cases was described in detail in our previous study [16]. After excluding patients who had not received any chemotherapy (N = 793), 1,078 patients with GC having distant metastasis, newly diagnosed in 2010 and who had received palliative first-line chemotherapy, were identified. Of these, 509 patients had received second-line chemotherapy. Lastly, 229 patients who had received third-line chemotherapy were identified as the final target population of this study (Fig 1).

From the HIRA database, data on patient demographics, palliative chemotherapy (regimen, time of administration, and duration), and survival status (or the status at the last follow-up) were extracted. Since this study was performed using publicly released data and investigators were completely blind to the information related to personal identification, the approval of this study was waived by the Institutional Review Board (IRB) at Seoul National University Bundang Hospital (IRB study number: X-1802-453-901).

### Statistical analysis

OS was defined as the interval from the start of third-line chemotherapy to the date of death or last follow-up. As of October 31, 2013 (the date of data cutoff), a patient who died of any cause or who was lost to follow-up for more than 1 year without further information on the survival status (i.e., if the last follow-up date was before November 1, 2012) was regarded as an event case. If a patient visited a hospital or clinic at least once within 1 year before October 31, 2013, and the survival status was not reported as dead in the last claim specification, the case was regarded as a censored case. The survival analysis was performed using the Kaplan-Meier method, and the log-rank test was used to compare the differences in OS between groups using univariable analysis. For multivariable analysis, a Cox proportional hazards regression model was used to estimate the adjusted hazard ratio (HR) in order to determine the significance of specified clinical variables on OS. All analyses were carried out using SAS/STAT software (version 9.4; Cary, NC, USA, SAS Institute Inc.) and IBM SPSS Statistics (version 18; Armonk, NY, USA, IBM Corp.). The statistical significance was set at *p* < 0.05.

## Results

### Patient characteristics

The study cohort consisted of 229 patients newly diagnosed in 2010 with distant metastasis, who had received palliative third-line chemotherapy. The baseline patient characteristics are summarized in Table 1. Group A (N = 229) includes all patients who had received third-line chemotherapy. The majority of patients (90.0%) were < 70 years of age, with a median age of 54 years (range, 25–82). Approximately 69% of patients were male. More than 90% of patients had received fluoropyrimidine and platinum during previous rounds of chemotherapy, and 43.7% and 40.6% of patients had previously received taxane and irinotecan, respectively. A variety of third-line chemotherapy regimens were prescribed and were classified as follows: taxane monotherapy (docetaxel or paclitaxel), taxane-based combination therapy, irinotecan monotherapy, irinotecan-based combination therapy, oxaliplatin plus 5-fluorouracil (FOLFOX), and others. Of these, irinotecan plus 5-fluorouracil doublet therapy was the most frequently prescribed regimen (37.1%). As is common clinical practice in most countries, including Korea, patients with MGC receive fluoropyrimidine (F) plus platinum (P) as first-line chemotherapy followed by taxane (T)-based or irinotecan (I)-based regimens as second-line chemotherapy. Therefore, we further subdivided the patients who had received F, P, and T followed by third-line I-based therapy and those who had received F, P, and I followed by third-line T-based therapy into Group B (N = 114). The patient characteristics in Group B were similar to those in Group A.

**Table 1:** Patient characteristics.

Parameters	Group A (N = 229)	Group B (N = 114)
**Age**		
< 70 years	206 (90.0%)	109 (95.6%)
≥ 70 years	23 (10.0%)	5 (4.4%)
**Sex**		
Male	159 (69.4%)	75 (65.8%)
Female	70 (30.6%)	39 (34.2%)
**Previously exposed drugs**		
Fluoropyrimidine	227 (99.1%)	114 (100.0%)
Platinum	216 (94.3%)	114 (100.0%)
Taxane	100 (43.7%)	62 (54.4%)
Irinotecan	93 (40.6%)	52 (45.6%)
**3**^**rd**^**-line chemotherapy regimens**		
Docetaxel	25 (10.9%)	22 (19.3%)
Docetaxel + Cisplatin	15 (6.6%)	12 (10.5%)
Paclitaxel	11 (4.8%)	9 (7.9%)
Paclitaxel + Cisplatin	9 (3.9%)	7 (6.1%)
Irinotecan	10 (4.4%)	6 (5.3%)
Irinotecan + 5-fluorouracil	85 (37.1%)	54 (47.4%)
Oxaliplatin + 5-fluorouracil	33 (14.4%)	-
Others	41 (17.9%)	4 (3.5%)

Group A: All patients who received third-line chemotherapy (N = 229)

Group B: Patients who received fluoropyrimidine (F), platinum (P), and taxane (T) followed by third-line irinotecan (I)-based therapy and patients who received F, P, and I followed by third-line T-based therapy (N = 114)

### Clinical parameters related to survival outcomes in all patients (Group A, N = 229)

**Fig 2:**
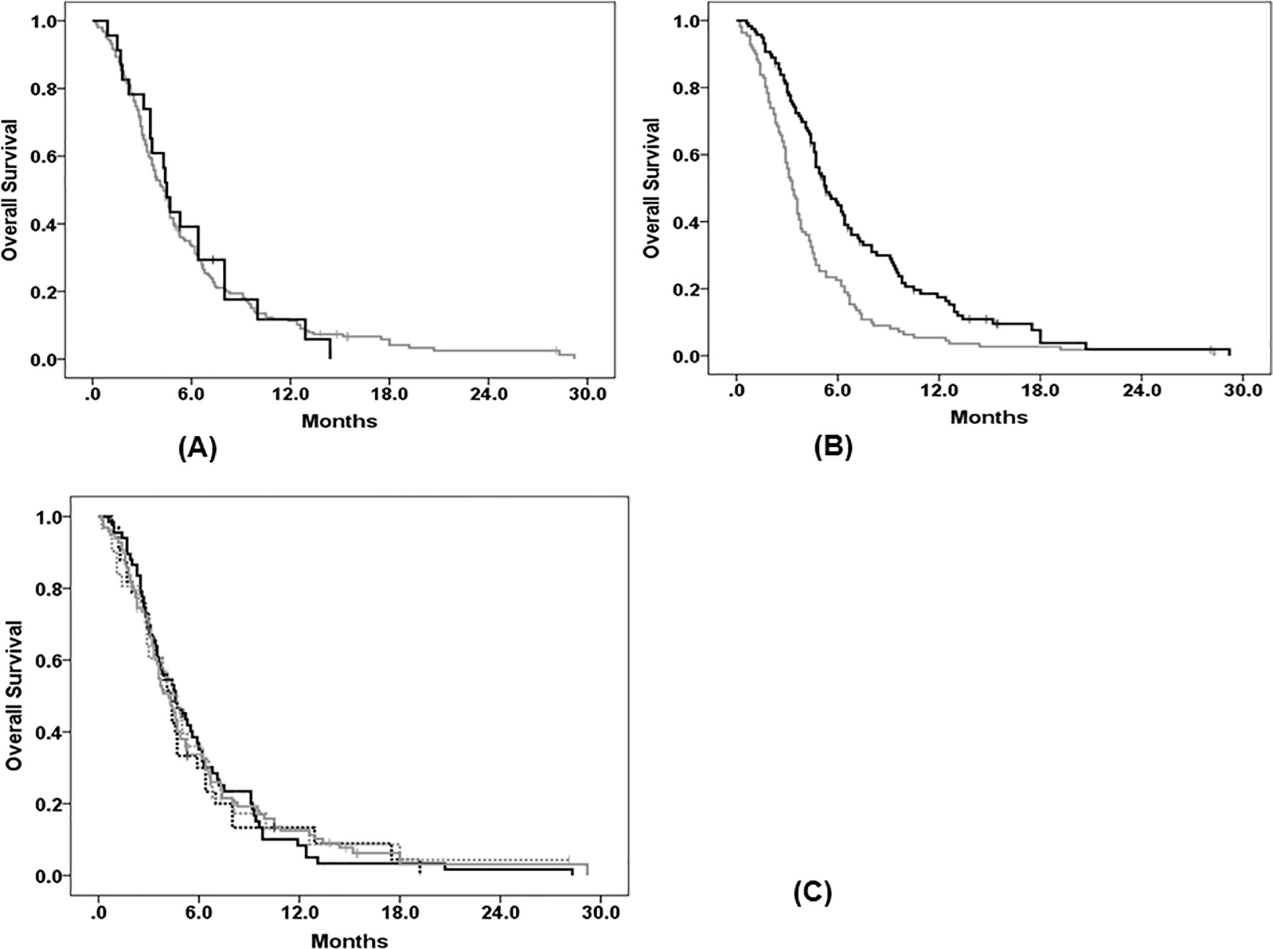
Kaplan–Meier survival curves for overall survival in the entire population (Group A, N = 229). (A) Survival curves for overall survival according to age. (B) Survival curves for overall survival according to chemotherapy regimens. (C) Survival curves for overall survival according to the median time from the start date of first-line chemotherapy to the start date of third-line chemotherapy (TF1T3).

The median OS of all 229 patients receiving third-line chemotherapy was 4.4 months (95% confidence interval [CI], 3.9–4.9) from the initiation of third-line chemotherapy. Age and gender were not significantly associated with OS (Table 2, Fig 2A). When we classified the chemotherapy regimens into four groups (T-based therapy, I-based therapy, FOLFOX, and others), the chemotherapy regimen was not a significant prognostic factor for OS in both univariable and multivariable analyses (Table 2, Fig 2B). In addition, there was no significant difference in OS between monotherapy and combination therapy (T-based monotherapy vs. T-based combination therapy; I-based monotherapy vs. I-based combination therapy); however, due to the small number of patients in these groups, the results need to be verified (S1 Fig). The median time from the start date of first-line chemotherapy to the start date of third-line chemotherapy (TF1T3) was 9.5 months (range, 1.5–36.6 months). The median OS of patients with a TF1T3 of ≥ 9.5 months was 5.3 months, compared to 3.3 months in patients who had a TF1T3 of < 9.5 months (log-rank test; *p* < 0.001) (Table 2, Fig 2C). Multivariable analyses showed that a longer TF1T3 was the only factor associated with a significant reduction in the risk of death (TF1T3 ≥ 9.5 months vs. < 9.5 months; HR, 0.53; 95% CI, 0.40–0.70).

**Table 2:** Clinical parameters related to survival outcomes in all patients (Group A, N = 229).

		Univariable analysis			Multivariable analysis	
	N	Overall survival (months; median)	*p*	Hazard ratio	95% confidence interval	*p*
Sex			0.676			0.873
Male	159	4.3	-	1.00	-	-
Female	70	4.6	-	0.98	0.73–1.31	-
Age (year)			0.875			
< 70	206	4.3	-	1.00	-	-
≥ 70	23	4.5	-	1.07	0.67–1.71	-
Duration from first-line to third-line chemotherapy			< 0.001			< 0.001
< 9.5 months (median)	111	3.3	-	1.00	-	-
≥ 9.5 months (median)	118	5.3	-	0.53	0.40–0.70	-
Chemotherapy regimens			0.985			0.961
FOLFOX	33	4.3	-	1.00	-	-
Taxane-based therapy	67	4.6	-	1.04	0.67–1.62	0.863
Irinotecan-based therapy	98	4.2	-	0.98	0.65–1.47	0.909
Others	31	4.7	-	0.92	0.55–1.55	0.762

### Clinical parameters related to survival outcomes in Group B (N = 114)

**Fig 3:**
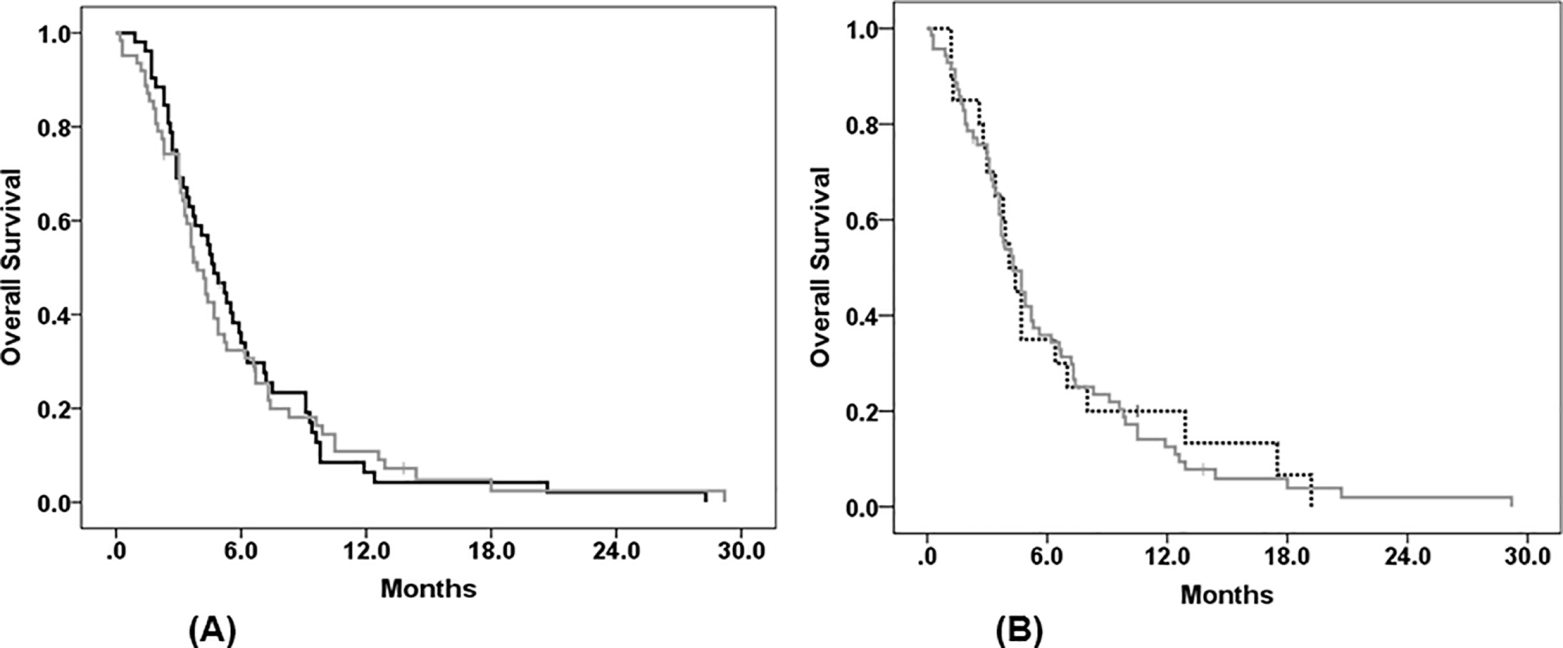
Kaplan–Meier survival curves for overall survival. (A) Survival curves for overall survival according to chemotherapy regimens in Group B (N = 114). (B) Survival curves for overall survival of patients receiving FOLFOX (N = 20) vs. taxane- or irinotecan-based chemotherapy as third-line therapy (Group B, N = 114).

Group B included patients who had received F, P, and T followed by third-line I-based therapy (N = 62) and those who received F, P, and I followed by third-line T-based therapy (N = 52). Clinical parameters related to survival outcome in Group B were similar to those in Group A. TF1T3 was the only factor significantly associated with OS in both univariable and multivariable analyses (Table 3). The median OS of patients who had received F, P, and T followed by third-line I-based therapy was similar to that of patients who received F, P, and I followed by third-line T-based therapy (3.9 months vs. 4.7 months, *p* = 0.894) (Fig 3A, Table 3). Therefore, the sequence of T and I did not impact on survival outcome. In addition, there was no significant difference in OS between monotherapy and combination therapy as the third-line treatment for MGC (T-based monotherapy vs. T-based combination therapy; I-based monotherapy vs. I-based combination therapy) (S2 Fig).

**Table 3:** Clinical parameters related to survival outcomes in Group B (N = 114).

		Univariable analysis			Multivariable analysis	
	N	Overall survival (months; median)	*p*	Hazard ratio	95% confidence interval	*p*
Sex			0.924			0.754
Male	75	4.4	-	1.00	-	-
Female	39	3.8	-	1.07	0.71–1.61	-
Age (year)			0.779			0.843
< 70	109	4.4	-	1.00	-	-
≥ 70	5	3.5	-	0.91	0.36–2.32	-
Duration from first-line to third-line chemotherapy			0.003			0.004
< 9.5 months (median)	59	3.3	-	1.00	-	-
≥ 9.5 months (median)	55	5.5	-	0.56	0.37–0.83	-
Chemotherapy regimens			0.894			0.808
Taxane-based therapy	52	4.7	-	1.00	-	-
Irinotecan-based therapy	62	3.9	-	0.95	0.64–1.41	-

### Efficacy of FOLFOX regimen as third-line chemotherapy

There are limited data on the efficacy of oxaliplatin in patients with MGC who were previously treated with cisplatin. In our study, 20 patients had received FOLFOX as third-line chemotherapy after previously receiving F, cisplatin, and T therapy or F, cisplatin, and I therapy. The median OS of FOLFOX-treated patients and that of patients in Group B (N = 114) was 4.1 months and 4.3 months, respectively (*p* = 0.679) (Fig 3B). Multivariable analysis indicated that chemotherapy regimen (FOLFOX vs. T- or I-based therapy) was not a significant factor related to OS (S1 Table).

## Discussion

To date, only a few studies have focused on third-line chemotherapy; therefore, data on third-line chemotherapy in patients with metastatic or recurrent GC are inconclusive [17–21]. In addition, the majority of these studies have been small phase II studies or retrospective studies that have evaluated the efficacy and safety of monotherapy or combinations of several cytotoxic agents. One study using various regimens as the third-line therapy reported a median PFS and OS of 2.6 and 6.4 months, respectively, and a response rate of 10.3% [17]. In small phase II studies of paclitaxel [18] or docetaxel [19, 20], response rates were in the range of 15–23%, with a median OS of 4–7 months. In another retrospective study of 158 patients, Kang et al. reported the efficacy and safety of third-line irinotecan and 5-fluorouracil (FOLFIRI) combination chemotherapy: median PFS and OS were 2.1 and 5.6 months, respectively, with tolerable toxicity profiles [21]. In real-world clinical practice, some patients who have a good PS after first- and second-line failure continue on to receive third-line chemotherapy; both F, P, and T followed by third-line I-based therapy or F, P, and I followed by third-line T-based therapy are common clinical practice patterns.

Recently, Kang et al. [22] reported a randomized phase III trial comparing nivolumab (programmed cell death protein 1 (PD-1) receptor inhibitor) with a placebo in 493 Asian patients with metastatic or recurrent GC who had received at least two prior regimens. Nivolumab significantly prolonged median OS from 4.14 to 5.26 months in this population (HR, 0.63; 95% CI, 0.51–0.78; *p* < 0.0001). Survival rates at 12 months were 26.2% with nivolumab compared to 10.9% with a placebo. Based on these results, administration of an active and tolerable therapy in the third-line setting may have a beneficial effect in patients with metastatic or recurrent GC; therefore, third-line therapy needs to be further investigated in future studies.

In this study, we conducted a nationwide population-based study reflecting real-world treatment patterns and outcomes in MGC patients receiving third-line chemotherapy. Of all the MGC patients newly diagnosed in 2010, approximately 21% had received third-line chemotherapy with various regimens containing taxane, irinotecan, or oxaliplatin. The median OS for all 229 patients receiving third-line chemotherapy was 4.4 months. Prognostic factor analyses for survival outcomes revealed that TF1T3 was the only factor significantly associated with OS, while age and gender had no impact on OS. In a report of FOLFIRI as third-line chemotherapy, TF1T3 was reported to be independently related to survival outcome [21]. To the best of our knowledge, no prospective analyses have been performed to evaluate prognostic factors in the third-line setting; therefore, we consider that TF1T3 could be particularly helpful in selecting patients who may benefit from third-line chemotherapy. Furthermore, TF1T3 needs to be included as a stratification factor when designing prospective studies evaluating third-line therapy in patients with metastatic or recurrent GC.

No significant difference in OS was observed between the patients who had received F, P, and T followed by third-line I-based therapy and those who had received F, P, and T followed by third-line I-based therapy, indicating that the sequence of chemotherapeutic agents does not affect OS. These results further suggest that both T and I are valid treatment options for salvage therapy in patients with metastatic or recurrent GC, and the main consideration for selecting salvage chemotherapeutic agents should be the previous therapy and side effects of each regimen as well as PS of patients.

Lastly, we evaluated the efficacy of FOLFOX as third-line chemotherapy. Since most patients with metastatic or recurrent GC are initially treated with fluoropyrimidine plus platinum, we usually do not include platinum in a salvage regimen for these patients. However, some reports suggest that there are benefits to introducing oxaliplatin after receiving cisplatin-containing chemotherapy in metastatic or recurrent GC [23–25]. In our study, the median OS of patients who had received FOLFOX as third-line chemotherapy was shown to be similar to that of patients who had received third-line T- or I-based therapy. Therefore, in concordance with previous reports [23–25], our data suggest that oxaliplatin has no cross-resistance with cisplatin and that some patients with metastatic or recurrent GC can benefit from FOLFOX chemotherapy in a third-line setting even after treatment failure with cisplatin.

There are several limitations in this study. First, we could not evaluate known prognostic factors for survival outcome, such as PS of patients and burden of metastasis, since the HIRA database does not collect information on these clinical variables. Second, in survival analysis, the exact survival status could not be retrieved from the database in 36.2% of patients (N = 83). We assumed that patients with no claim submitted by hospitals/clinics within 1 year before the data cutoff date must have died of MGC or another cause, considering the natural course of MGC patients after failure to second-line chemotherapy. For these patients, we considered the last date of their visit to the hospitals/clinics as the date of death; therefore, the survival duration might be underestimated in some patients. However, as most Korean patients die in hospital rather than at home and all patients’ visits to hospital/clinics are recorded on the HIRA database, the difference between the date of the last visit to hospitals/clinics and the real date of death is thought to be very short, if any [16]. Third, because the selection process of patients was based on strict inclusion criteria, only 229 patients were finally selected even though this was a nationwide population-based study. To select MGC cases more precisely, we excluded gastrectomy cases with the diagnostic codes of distant metastasis or recurrence cases after prior curative gastrectomy [16]. Therefore, only MGC with de novo metastatic disease and without gastrectomy were included, which might explain the shorter median OS in our patient population (4.4 months) than the OS reported in previous studies [17–22]. In addition, most patients with stage II or III disease after primary gastrectomy with D2 lymph node dissection receive adjuvant fluoropyrimidine with or without platinum chemotherapy in Korea; and so, if we included recurrence cases after prior curative gastrectomy, the results might be somewhat different.

Nevertheless, our study has importance. This was a population-based study evaluating treatment patterns and outcomes in MGC patients receiving third-line chemotherapy and therefore, it is considered to reflect the real-world clinical situation of third-line chemotherapy. As there is limited evidence to guide decisions on third-line chemotherapy in patients with MGC, this analysis will provide a better understanding of these patients and help guide clinical care in real-world clinical practice.

To summarize, in this population-based study we observed that approximately 21% of all MGC patients had received third-line chemotherapy. The median OS of these patients was 4.4 months, and a longer TF1T3 was associated with an increased OS after the initiation of third-line chemotherapy. In addition, the sequence of using T and I as subsequent therapy after first-line failure was not shown to impact on survival outcome. Overall, this study will help guide clinical care of this population as well as design prospective clinical trials for these patients.

## Supporting information

S1 Fig Kaplan–Meier survival curves for overall survival in the entire population (Group A, N = 229) according to various chemotherapy regimens. (TIF)

S2 Fig Kaplan–Meier survival curves for overall survival of patients receiving monotherapy vs. combination therapy (Group B, N = 114). (TIF)

S1 Table Clinical parameters related to survival outcomes (FOLFOX vs. taxane-or irinotecan-based chemotherapy, N = 134). (DOCX)
