# Supplementary figures and images for: Treatment patterns and outcomes in patients with metastatic gastric cancer receiving third-line chemotherapy: A population-based outcomes study
Source: PLoS One. 2018 Jun 7;13(6):e0198544. doi: 10.1371/journal.pone.0198544 (PMC5991719; doi:10.1371/journal.pone.0198544)

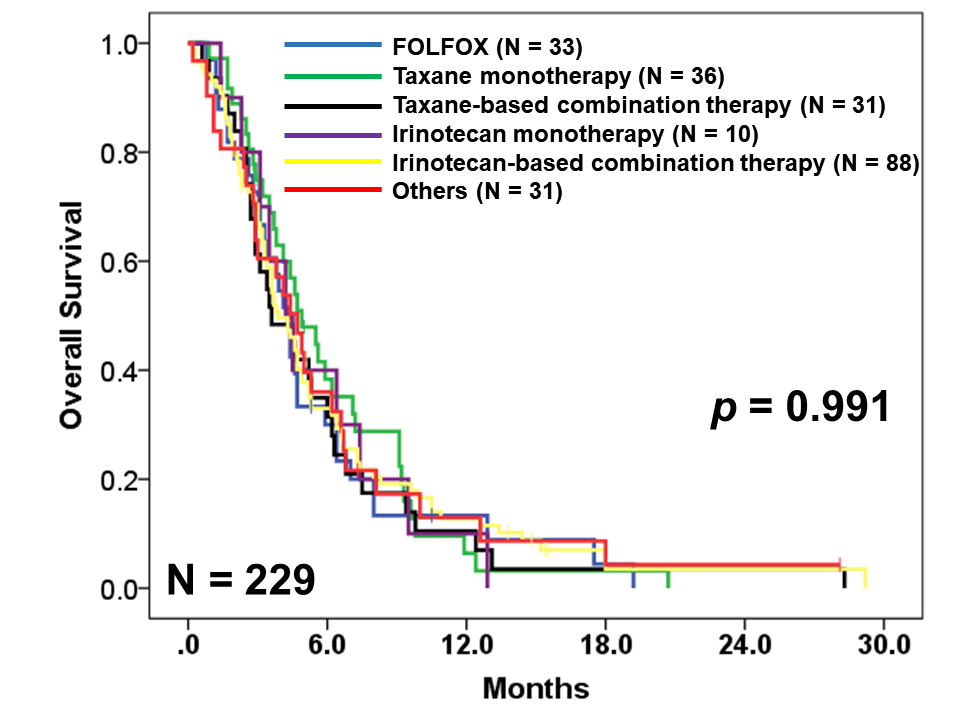

Supplement: S1 Fig — (TIF) [file pone.0198544.s001.TIF]

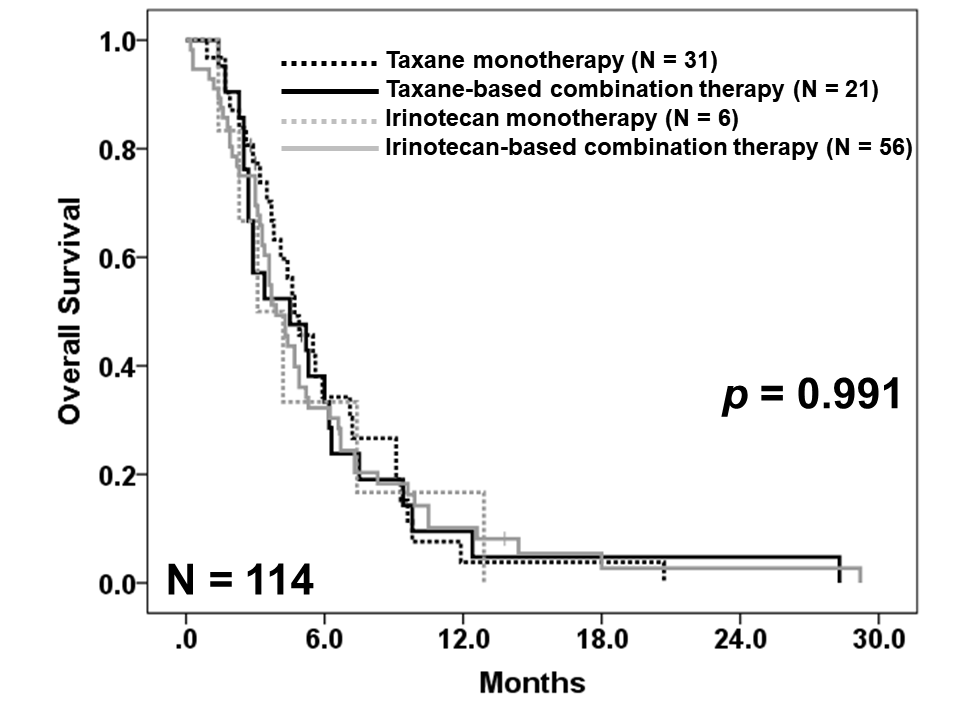

Supplement: S2 Fig — (TIF) [file pone.0198544.s002.TIF]
