# Supplementary material for: Treatment patterns and outcomes in patients with metastatic gastric cancer receiving third-line chemotherapy: A population-based outcomes study
Source: PLoS One. 2018 Jun 7;13(6):e0198544. doi: 10.1371/journal.pone.0198544 (PMC5991719; doi:10.1371/journal.pone.0198544)
Supplement: S1 Table — (DOCX) [file pone.0198544.s003.docx]

**S1 Table.** Clinical parameters related to survival outcomes (FOLFOX vs. taxane-or irinotecan-based chemotherapy, N = 134)

|  |  |  | Univariable analysis |  |  |  | Multivariable analysis |  |
| --- | --- | --- | --- | --- | --- | --- | --- | --- |
|  | N |  | Overall survival  (months; median) | *p* |  | Hazard ratio | 95% confidence interval | *p* |
| Sex |  |  |  | 0.927 |  |  |  | 0.714 |
| Male | 92 |  | 4.4 | - |  | 1.00 | - | - |
| Female | 42 |  | 3.8 | - |  | 1.08 | 0.73-1.59 | - |
| Age (year) |  |  |  | 0.695 |  |  |  | 0.713 |
| < 70 | 126 |  | 4.3 | - |  | 1.00 | - | - |
| ≥ 70 | 8 |  | 3.6 | - |  | 0.87 | 0.42-1.82 | - |
| Duration from first-line to third-line chemotherapy |  |  |  | 0.002 |  |  |  | 0.002 |
| < 9.5 months (median) | 67 |  | 3.4 | - |  | 1.00 | - | - |
| ≥ 9.5 months (median) | 67 |  | 5.3 | - |  | 0.57 | 0.40-0.82 | - |
| Chemotherapy regimens |  |  |  | 0.679 |  |  |  | 0.936 |
| Taxane- or irinotecan-based  therapy | 114 |  | 4.3 | - |  | 1.00 | - | - |
| FOLFOX | 20 |  | 4.1 | - |  | 0.98 | 0.59-1.63 | -- |
